# Smart Home Control and Management Based on Big Data Analysis

**DOI:** 10.1155/2022/3784756

**Published:** 2022-02-10

**Authors:** Hao Chi, Yuyan Chi

**Affiliations:** ^1^College of Information Engineering Press, Shandong Vocational and Technical University of International Studies, Rizhao, Shandong 276800, China; ^2^Huilin Training, Shandong Vocational and Technical University of International Studies, Rizhao, Shandong 276800, China

## Abstract

In order to improve the effect of smart home control and management, a new smart home control and management method based on big data analysis is designed. The basic hardware of smart home control and management is designed, including smoke sensor hardware, temperature and humidity sensor hardware, and infrared sensor hardware, so as to collect smart home data and realize data visualization and buzzer alarm. The collected data are transmitted through the indoor wireless network of smart home gateway equipment, and the data distributed cache architecture based on big data analysis is used to store smart home data. Based on the relevant data, the hybrid particle swarm optimization algorithm is used to schedule the control and management tasks of smart home to complete the control and management of smart home. The experimental results show that the device control and scenario management effect of this method is better, and the communication performance is superior and has high practical application value.

## 1. Introduction

The increasing development of information technology and control technology has gradually accelerated the pace of social informatization and also promoted the informatization of people's way of life, work, and communication [1, 2]. The high development of information technology, liberalization, and high level of communication have not only changed people's daily living habits, but also posed a challenge to traditional housing. The expanding material demand makes people's requirements for home no longer a simple material space, but pay more attention to a beautiful, convenient, comfortable, and safe living environment and advanced communication facilities, efficient and simple terminal control equipment, and automatic and intelligent network management of household appliances and resources [3]. Due to the trend of high informatization in modern society, people eagerly hope that all household equipment in the residential environment can be added to the Internet. With the deep integration of hardware and software, the smart home control system has fully met people's needs, because it networks all household appliances and building intelligence, which not only meets the exchange of internal environment and external information, but also realizes a safe, comfortable, and convenient lifestyle [4–6].

Smart home is a popular topic in today's era. It is one of the specific manifestations of the Internet of Things [7]. It is to deeply integrate Internet of Things technology, wiring technology, network security technology, intelligent control technology, wireless communication technology, and other technologies; connect various hardware facilities in the house; and then control household appliances, lighting equipment, security and antitheft, and environmental detection, so as to establish an intelligent management system and to facilitate people's daily life [8]. The use of a smart home system in the environment with residence as the platform not only optimizes and facilitates people's lifestyle, but also increases the beauty of life and the safety performance of people's life. After entering the new era, with the great development of information technology, people's traditional ideas have also changed greatly, so their understanding of housing is becoming deeper and deeper [9, 10].

In the future information-based life of mankind, the formation of a smart home environment has become an inevitable trend. In the future of smart home development, relevant national functional institutions and departments should also provide a lot of support and encouragement, so that the application prospect and market of smart home will be clear [11, 12]. According to the current situation, the development of smart home in China is still in the primary stage, whether at the technical level or theoretical level. For example, the unified specification of smart home technology has not been formed, so that many different products cannot be compatible, and the availability is still relatively low, which brings a lot of inconvenience to the user experience and manufacturers' production. However, due to its high design cost, many ordinary people are deterred. At the same time, the traditional smart home has complex wiring. The function of the collection is relatively single, and the traditional smart home control and management scheme are incomplete, which are urgent problems to be solved [13].

Therefore, this article designs a new smart home control and management method based on big data analysis.

## 2. Smart Home Control and Management

### 2.1. Basic Hardware Design of Smart Home

#### 2.1.1. Smoke Sensor Hardware Circuit

The smoke concentration sensor is specially used to observe whether the smoke concentration in the kitchen exceeds the safe range. Therefore, this article designs to use the smoke sensor MQ2 to detect the smoke concentration. The gas sensor has high sensitivity and adjustable sensitivity, and can respond to most smoke [14], especially for methane (CH4), the main component of natural gas used in the family.  Table 1 shows the detection range of some smoke by MQ2.

**Figure 1:**
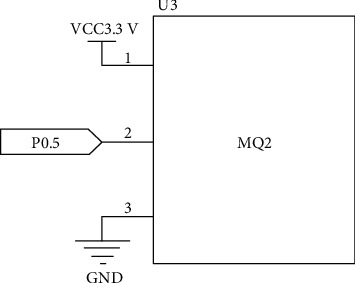
MQ2 circuit connection diagram.

The smoke sensor module MQ2 selects digital output. That is, when the indoor smoke concentration exceeds the threshold, MQ2 module will output low level, and then, the buzzer will give an alarm. If the smoke concentration does not exceed the threshold, the MQ2 module will output high level [15, 16]. The MQ2 pin of the module is connected with pin P0.5 of CC2530 chip, so the mechanism of detecting high and low levels is used to detect whether the concentration of harmful gas smoke in the environment exceeds the standard value. The MQ2 circuit connection diagram is shown in Figure 1.

#### 2.1.2. Hardware Circuit of Temperature and Humidity Sensor

In this article, DHT11 temperature and humidity sensor is used to collect the temperature and humidity in the house. It is a composite sensor that can collect both temperature and humidity [17]. The pin description of DHT11 is shown in Table 2.

**Figure 2:**
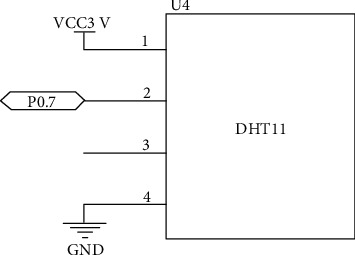
DHT11 circuit connection diagram.

The connection circuit diagram according to Table 2 is shown in Figure 2.

#### 2.1.3. Infrared Sensor Hardware Circuit

**Figure 3:**
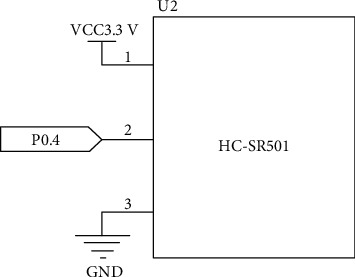
Circuit connection diagram of HC-SR501.

The infrared sensor module HC-SR501 is used to detect whether there are people close to the house. It can detect the infrared radiation from the human body. The high and low levels are the digital signals [18–20]. If someone steps into the sensing range of the infrared sensor, the output terminal will send a high level, and the output terminal will output a low level after the person leaves.  Figure 3 shows the circuit connection diagram of the module.

The characteristic parameters of the infrared sensor are shown in Table 3.

#### 2.1.4. Buzzer Alarm Circuit

**Figure 4:**
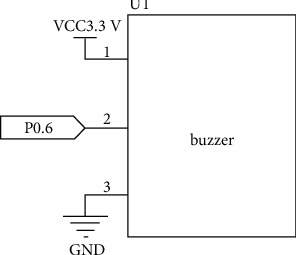
Circuit diagram of buzzer connection.

The buzzer alarm circuit module is driven by S8550 triode, and the working voltage is 3.3 V–5 V. When the temperature and humidity exceed the preset value, the IO port P0.6 of the ZigBee module will output low level, and the buzzer will sound. At the same time, when toxic gas appears in the environment and someone approaches the house, the buzzer will also sound.  Figure 4 is the connection circuit diagram of the buzzer.

#### 2.1.5. LCD Module

**Figure 5:**
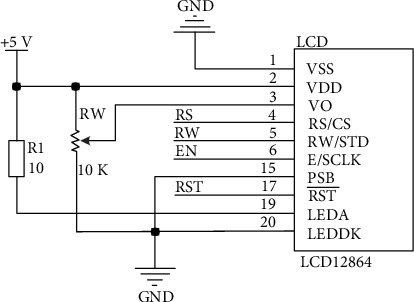
Circuit diagram of liquid crystal display.

This article adopts Shenzhen Qinchuangjia 12864 display screen. The controller used in the display screen is ST7920, which supports two driving modes of serial port and parallel port. Due to the limited STM32 pins used in the method design in this article and the large number of IO ports in the parallel port mode, the requirements for the screen refresh rate, that is, speed, are not high. Therefore, the serial port driving mode (PSB pin connected to GND) is adopted in this article, and the schematic diagram is shown in Figure 5.

### 2.2. Indoor Wireless Networking Design of Smart Home Gateway Equipment

The most basic function of smart home gateway equipment is indoor networking, which enables home terminal equipment to connect to gateway equipment through home intranet. Users use client equipment to control home terminal equipment through gateway equipment. This article mainly puts forward and analyzes the hardware structure of the designed home gateway device [21–23], specifies the format of data packet, and develops and designs the intranet part of the gateway combined with Bluetooth, ZigBee, and WiFi communication modules.

**Figure 6:**
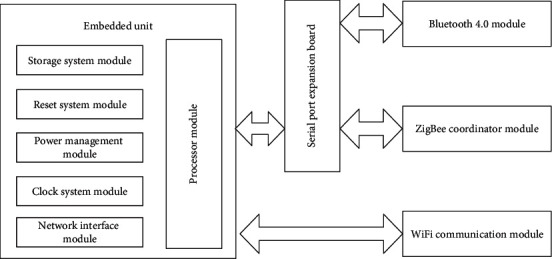
Hardware structure of smart home gateway.

The hardware part of smart home gateway device is mainly composed of embedded unit based on ARM architecture, serial port expansion board, Bluetooth, ZigBee [24, 25], and WiFi wireless communication module. The hardware structure of smart home gateway device is shown in Figure 6.

As can be seen from Figure 6, the embedded unit is first connected with the serial port expansion board, and the serial port expansion board is then connected with Bluetooth and ZigBee modules. The embedded unit is directly connected with the WiFi communication module [26].

As one of the wireless communication modules, the Bluetooth module is mainly aimed at Bluetooth devices in smart home, such as Bluetooth audio, Bluetooth headset, and smart bracelet; these kinds of smart home devices are generally configured flexibly and can be increased or decreased according to needs at any time [27, 28].

ZigBee module is mainly used as coordinator equipment at the gateway of the smart home network and is the central part of the whole ZigBee network [29, 30]. ZigBee's devices are mainly relatively fixed devices with low transmission speed requirements, such as smart home devices such as lighting and environmental detection.

WiFi communication module is also one of the wireless communication modules. WiFi is the most widely used wireless communication mode at this stage. With the popularity of mobile phones, tablets, and other devices, using such devices as client devices of the smart home system can easily control home terminal devices. At the same time, the fast transmission speed of WiFi is very suitable for data transmission on multimedia smart home devices such as video.

The serial port expansion board is mainly used to connect various wireless communication device modules, preliminarily sort out the information collected by the wireless device, and transmit it to the embedded unit through the serial port. At the same time, the control instructions transmitted from the embedded unit are transmitted to the corresponding wireless communication module according to different kinds of wireless communication requirements, and then transmitted to the home intelligent terminal device to complete the communication.

The embedded unit is the core of the whole smart home gateway equipment. It is responsible for gateway authentication, data analysis, processing, storage, and data exchange between internal and external networks. It is developed based on OpenWrt embedded system [31].

The operation process of the smart home system mainly includes two parts: data transmission and data processing. Data processing can only be completed after receiving data normally, so the stability of data transmission process is particularly important. Only normal data transmission can ensure the stable operation of the whole smart home system. The design of data transmission protocol includes two points: the first point is the process design of data transmission, mainly including the direction of data transmission and the transceiver of data transmission; The second point is the data packet communication format used in the data transmission process. A reasonable data packet communication format can ensure the target of data transmission and the correctness of communication data, and ensure the stable operation of relevant equipment.

#### 2.2.1. Data Stream Transmission

**Figure 7:**
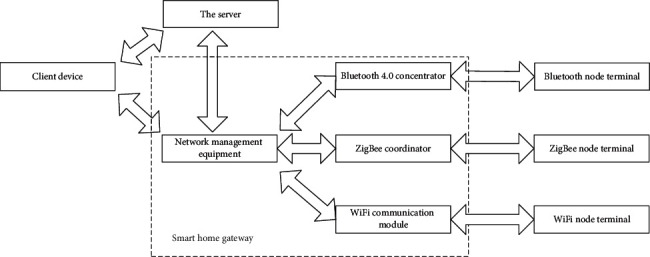
Data stream transmission diagram of smart home.

The data information flow generated in the operation of smart home mainly includes two types: control data information flow and state data information flow. The control data information flow refers to the control instructions sent by the smart client device to the home terminal device [32, 33]; Status data information flow refers to the data information that the home terminal device transmits its own status information and collected environmental information to the smart client device, and then feeds back to the user through the interactive interface. Smart home data stream transmission is shown in Figure 7.

#### 2.2.2. Packet Format Definition

**Figure 8:**
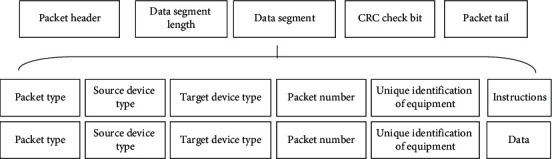
Communication packet structure.

Packet format is one of the key factors affecting the normal transmission of data. The data packet with perfect format can ensure the integrity of data in the transmission process, reduce the operation load, and improve the stability and security of related equipment. The data packet includes five parts: packet header, data segment length, data segment, CRC check, and packet tail. The data packet designed in this article is shown in Figure 8.

When errors are found in the data transmission process, the data packet can be tracked by combining the data packet type, source device type, target device type, data packet number, and device unique identification to find out the causes of data abnormalities and realize the self-inspection function of transmission status in the transmission process [34]. At the same time, defining traceable data packets also facilitates function development and improves the intelligence of smart home control and management.

### 2.3. Data Distributed Cache Architecture Based on Big Data Analysis

File storage system is the cornerstone of the whole smart home, which is very important for the efficient and smooth operation of related devices. This chapter mainly studies the data generated in the operation of smart home and analyzes the data to be stored, such as images and videos. Among them, video data are relatively large and can be directly stored in HDFS, and structured data such as video can be stored in distributed database HBase [35, 36]. At present, there are some problems in the distributed cache system and massive small file storage. This chapter designs a distributed cache system for caching hot data and a small file storage system for storing massive pictures.

**Figure 9:**
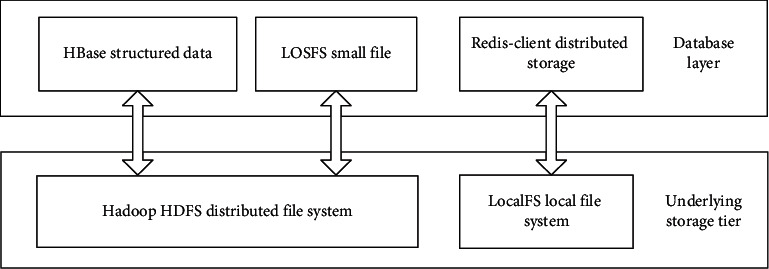
Module diagram of the file storage system.

The schematic diagram of file enclosure is shown in Figure 9.

1. HDFS is suitable for storing large files and can ensure high reading and writing speed, so video data are stored directly in HDFS. At the same time, HBase components are arranged on the HDFS cluster to store structured data.
2. Distributed cache. In order to improve the speed of applications accessing hotspot data, a cache needs to be built. Due to the limitation of single machine cache capacity, a distributed cache system is built to meet the high-capacity cache requirements of applications.
3. Massive small files. A large number of small files will be generated in the process of video processing, such as image data. The loss system is designed to store a large number of small files. It merges the small files, records the index information corresponding to each small file, and stores the merged files in HDFS.

Due to the processing performance and memory capacity limitations of a single machine, the cache system built with a single machine cannot meet the needs of high-capacity cache. At the same time, if a single machine is used to cache data, once the machine fails, the whole cache will fail, which will have an extremely adverse impact on the application. Therefore, it is necessary to use multiple machines to build a distributed cache system, and each machine is responsible for the storage and processing of some cached data, so that the cache system has large storage capacity and strong processing ability. Redis is widely used to build the cache system. Redis is an efficient key-value pair data storage system based on memory, which is written in C language. It supports the construction of a distributed cache system. The official Redis cluster is designed based on centerless and intelligent end. Redis-client must send the request directly to the corresponding node of the cluster according to the hash of the key, which means that it must be responsible for handling complex jump logic, which makes it difficult to write programs, and different Redis nodes in the cluster are highly coupled, which makes it difficult to upgrade Redis.

**Figure 10:**
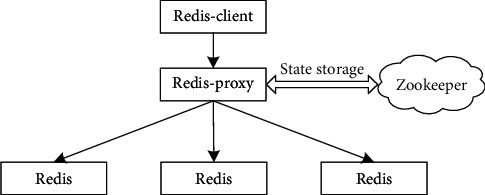
Schematic diagram of distributed cache architecture.

Based on the above analysis, this article takes Redis as the basis, and before Redis-proxy, Redis-client operates the cached data by connecting Redis-proxy. Redis-proxy will distribute the request to the corresponding node according to the key in Redis command and forward the processing results to Redis-client. At the same time, the use method of Redis-proxy is the same as that of single Redis, which solves the problem of complex Redis-client processing logic in the official Redis cluster.  Figure 10 is the schematic diagram of distributed cache architecture.

The specific scheme is to add a Redis-proxy in front of Redis, which will receive the request as an agent. When the agent receives the request, Redis-proxy calculates the data storage node according to the key in the command, forwards the request to the corresponding service node, summarizes the results after processing, and forwards the results to Redis-client. Redis-proxy has no status and is easy to expand. At the same time, the underlying storage engine is still Redis itself, which uses zookeeper to store the distribution status of cached data in the cache system. For the upper application, there is no difference between connecting to Redis-proxy and connecting to the native Redis server. Request forwarding will be carried out at the bottom, but it is transparent to Redis-client.

The distributed cache system uses prepartitioning to manage cached data. By default, the cache is divided into 1024 slots. The slot numbers corresponding to data keys are determined based on formula (1). Formula (1) uses CRC32 to calculate the CRC32 check code corresponding to the crC32 key. Compared with MD5, CRC has the advantages of simple implementation and fast calculation. Therefore, CRC is selected for data hashing. The slot number is determined as follows:

(1) $$\begin{array}{c} \text{ID}=\text{scr}32\left(\text{key}\right)\%1024. \end{array}$$

**Figure 11:**
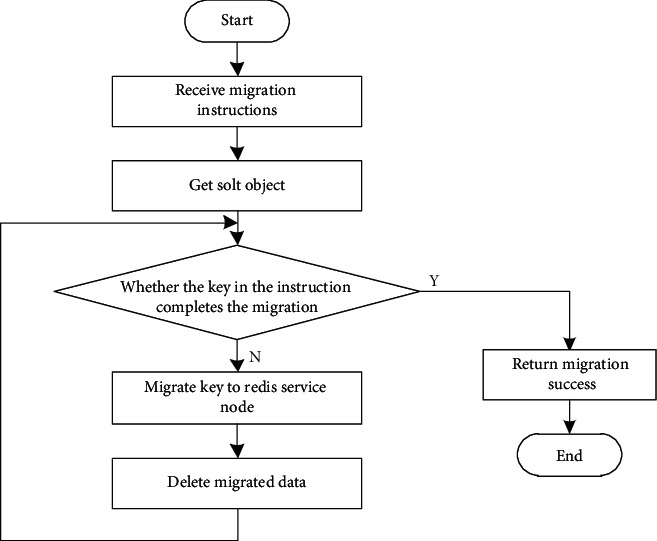
Migration process.

After receiving the migration instruction, the Redis service node establishes a TCP connection with the destination Redis service node for the transmission of migration data and migrates all data under the slot specified in the migration instruction to the destination Redis service node. After the migration is completed, the data stored by itself will be deleted. The process is shown in Figure 11.

### 2.4. Task Scheduling Based on Hybrid Particle Swarm Optimization Algorithm

Smart home control and management task scheduling is very important for users. Task scheduling is a reasonable scheduling between different processing nodes and tasks to reduce task completion time and improve efficiency. However, task scheduling is a NP-complete combinatorial optimization problem, and the optimal solution cannot be obtained in polynomial time. Considering the superiority of particle swarm optimization (PSO) in scheduling optimization problem, the PSO algorithm is selected to find the relative optimal solution of task scheduling. Aiming at the problem that PSO is easy to fall into local optimization and convergence is too slow, a hybrid particle swarm optimization algorithm is designed and applied to smart home control and management task scheduling.

PSO algorithm is a random optimization algorithm, which is inspired by the social behavior of birds and fish. In the PSO algorithm, a group of individuals called particles fly in the search space, and each particle represents the candidate solution of the optimization problem. The current position of a particle is affected by the best position accessed by itself and the best position accessed by the whole population. The best position accessed by itself is called the historical best particle, and the best position accessed by the whole population is called the global best particle. According to different optimization problems, the position of each particle is evaluated by using the fitness function, and the particles are updated by using the following formulas until the result meets the set termination conditions or reaches the set maximum number of iterations.

(2) $$\begin{array}{c} v_{id}^{k+1}=\omega v_{id}^{k}+c_{1}R_{1}\left(p_{id}^{k}-x_{id}^{k}\right)+c_{2}R_{2}\left(p_{gd}^{k}-x_{id}^{k}\right), \end{array}$$

(3) $$\begin{array}{c} x_{id}^{k+1}=x_{id}^{k}+v_{id}^{k+1}, \end{array}$$

where *v*_*id*_^*k*+1^ and *x*_*id*_^*k*+1^ represent the current velocity and position of the particle when the number of population iterations is *k*, respectively; *p*_*id*_^*k*^ and *p*_*gd*_^*k*^ represent the historical best position of particles and the best position of particles in the whole population when the number of population iterations is *k*, respectively; *c*_1_ and *c*_2_ are weight factors, which are called cognitive learning factor and social learning factor, respectively; *R*_1_ and *R*_2_ are two random numbers with values between 0 and 1; and *ω* is the inertia weight coefficient, which is not only to avoid the infinite increase of particle velocity, but also a key parameter affecting the search results and convergence speed. During the iteration of the algorithm, *ω* will be dynamically adjusted. At the beginning of the iteration, a larger value of *ω* can make the algorithm have better global search ability. In the later stage of iteration, a smaller value of *ω* can improve the local exploration ability of the algorithm and carry out detailed search in a smaller local range. The following formular is an updated formula of *ω*:

(4) $$\begin{array}{c} \omega =\frac{\omega_{b}+\left(\omega_{e}-\omega_{b}\right)*k}{K_{\max}}. \end{array}$$

Among them, *ω*_*b*_ and *ω*_*e*_ are the initial value and end value of the iteration of the inertia weight coefficient, respectively, *k* is the current iteration times of the algorithm, and *K*_max_ is the maximum iteration times set by the algorithm.

In PSO, the size of *ω*, *c*_1_, and *c*_2_ is very important to the performance of the algorithm. Some relevant literature points out that when the three meet a certain relationship, the algorithm has convergence, but it cannot guarantee the convergence to the global optimal value. Using a random method to initialize particles may lead to particles gathering in a local area, which reduces the diversity of the population and is not conducive to the initial iteration of the population. With the increase in the number of population iterations, the particles will gradually tend to the global optimal position, and the population diversity will become worse, which may lead to the local optimization of the algorithm.

A new initialization method is designed, which uses the opposition-based learning method and chaotic system to generate the initial population of HPSO. The following is the calculation method of chaotic mapping:

(5) $$\begin{array}{c} ch_{g+1}=\sin\left(\pi ch_{g}\right),\\ ck_{g}\in \left(0,1\right),\\ g<G_{\max}, \end{array}$$

where *g* represents the iteration counter and *G*_max_ is the preset maximum chaotic iteration times. Since the value generated by chaotic mapping is between 0 and 1, it needs to be mapped to the solution space of the algorithm. The mapping method is shown in the following formula:

(6) $$\begin{array}{c} x_{j}=x_{\min,j}+ch_{j}\left(x_{\max,j}-x_{\min,j}\right). \end{array}$$

where *j* represents the dimension corresponding to the particle generated by chaotic mapping, *ch*_*j*_ is the mapping variable generated after *G*_max_ iterations of the chaotic system, *x*_max,*j*_ and *x*_min,*j*_ represent the lower and upper bounds of the *j*-th dimension of the particle, respectively, and and *x*_*j*_ is the value corresponding to the third dimension of the particle generated by chaotic mapping. After an initial particle is obtained through chaotic mapping, its opposite particle is obtained by using the opposite learning method. The calculation method is shown in the following formula:

(7) $$\begin{array}{c} x_{j}^{*}=x_{\min,j}+x_{\max,j}-x_{j},\\ x_{j}\in \left[x_{\min,j},x_{\max,j}\right]. \end{array}$$

In the HPSO algorithm, the learning method based on chaotic mapping and opposition is used to initialize the population, which ensures that the initial particles are evenly distributed in the search space, improves the diversity of the initial population, speeds up the convergence speed of the algorithm, and is conducive to the algorithm searching for the optimal solution.

Inertia weight is an important control parameter of the PSO algorithm. It can effectively control the global and local search ability. Therefore, it is necessary to design an appropriate inertia weight strategy to make the HPSO algorithm achieve the best balance between global search and local search. The inertia weight adjustment function used in HPSO is shown in the following formula:

(8) $$\begin{array}{c} \omega \left(k+1\right)=\omega_{\max}e^{-{\left(k/K\max\right)}^{2}}, \end{array}$$

where *ω*_max_ is the initial inertia weight, which is generally set to 0.9, 2 represents the current iteration times of the algorithm, and *k* is the maximum iteration times set by the algorithm. It can be seen that in the early stage of the HPSO algorithm, the inertia weight is large and the downward trend is slow, which makes the algorithm have better global search ability. In the later stage of the HPSO algorithm, the inertia weight is small and decreases rapidly, which makes the algorithm have better local search ability.

In the PSO algorithm, each particle adjusts its self-learning behavior by approaching the best particle experienced by the population, that is, part *c*_2_*R*_2_(*p*_*gd*_^*k*^ − *x*_*id*_^*k*^) in formula (2). Therefore, the update speed and efficiency of the best particle directly affect the whole population. In the process of evolution, we should not only consider the historical best position and global best position of particles, but also consider the evolution of the overall trend of particles:

(9) $$\begin{array}{c} \text{PSC}\left(k\right)=\sum_{i=1}^{S}R_{i}\left(k\right)*X_{i}^{k}. \end{array}$$

where *k* represents the current number of iterations, *X*_*i*_^*k*^ represents the *i*-th particle of the population, *S* represents the number of particles in the population, and *R*_*i*_(*k*) represents the ratio of the fitness of the *i*-th particle to the total fitness of all particles, which is given as

(10) $$\begin{array}{c} R_{i}\left(k\right)=\frac{f\left(X_{i}^{k}\right)}{\sum_{r=1}^{S}f\left(X_{i}^{k}\right)}. \end{array}$$

where *f*(*X*_*i*_^*k*^) represents the fitness of particle *i*. The larger its value, the closer the particle is to the optimal solution, and the larger *R*_*i*_(*k*), so the larger the proportion of its corresponding particles in PSC. PSC is introduced into the particle velocity update of the HPSO algorithm, and formula (2) is modified as shown in the following formula:

(11) $$\begin{array}{c} v_{id}^{k+1}=\omega v_{id}^{k}+c_{1}R_{1}\left(p_{id}^{k}-x_{id}^{k}\right)+c_{2}R_{2}\left(p_{gd}^{k}-x_{id}^{k}\right)+c_{3}R_{3}\alpha \left(PSC_{d}-x_{id}^{k}\right). \end{array}$$

The PSO algorithm loses diversity quickly at the initial stage of iteration, which makes the algorithm easy to fall into local optimization. Therefore, the HPSO algorithm uses formula (11) to update particle velocity as much as possible at the initial stage of iteration, so as to maintain population diversity. In the later stage of iteration, in order to reduce the amount of calculation, improve the calculation speed, and accelerate the convergence speed of the algorithm, formula (2) is used to update the particle speed. In order to achieve the above purpose, a function whose value decreases linearly with the increase in the number of iterations of the algorithm is designed, as shown in the following formula:

(12) $$\begin{array}{c} N_{k}=1-\frac{k}{K_{\max}}. \end{array}$$

At the later stage of the algorithm iteration, all particles in the particle swarm are guided by the global optimal particles and will gradually tend to the global optimal position. If the algorithm has found the optimal solution, all particles will gather near the optimal solution position, so that their fitness values tend to be equal. Therefore, the similarity of fitness values of all particles can be used to judge whether the algorithm finds the optimal solution. Specifically, it can be judged by the fitness variance of the population, as shown in the following formula:

(13) $$\begin{array}{c} \sigma^{2}=\frac{\sum_{i=1}^{S}\left(f\left(X_{i}^{k}\right)-\bar{f}\right)}{S}, \end{array}$$

where $\bar{f}$ represents the average fitness of all particles, and its calculation formula is as follows:

(14) $$\begin{array}{c} \bar{f}=\sum_{s=1}^{S}\frac{f\left(X_{i}^{k}\right)}{S}. \end{array}$$

*σ* ^2^ is the fitness variance of the population, which represents the aggregation degree of particles in the population. The smaller its value is, the more aggregated the particles in the population are. When its value is close to 0, it means that the fitness values of all particles in the population are close to the same, which indicates that the algorithm converges globally and finds the optimal solution. Therefore, a threshold *T* is set to determine the convergence of the algorithm when the fitness variance of the population is less than the set threshold. To sum up, the termination condition of the HPSO algorithm is that the variance of fitness of all particles in the population is less than the set threshold or reaches the maximum number of iterations set by the algorithm.

Task scheduling refers to allocating the most appropriate resources to the tasks to be executed under the consideration of different factors such as time, cost, and resource utilization, which can reduce the task completion time and improve the utilization of system resources.

HPSO-based smart home control management task scheduling mainly includes the following steps:

(1) Set parameters in the HPSO algorithm, including maximum iteration times, population size, initial inertia weight value, learning factor, and other parameters.
(2) Randomly generate a specified number of tasks, set the hardware configuration parameters of the virtual machine, and generate the ETC matrix:
  (15) $$\begin{array}{c} \text{ETC}=\left[\begin{array}{cccccccccc} 0 & 0 & 0 & 0 & 1 & 0 & 0 & 0 & 1 & 0\\ 0 & 0 & 1 & 0 & 0 & 0 & 1 & 0 & 0 & 1\\ 1 & 0 & 0 & 1 & 0 & 1 & 0 & 0 & 0 & 0\\ 0 & 1 & 0 & 0 & 0 & 0 & 0 & 1 & 0 & 0 \end{array}\right]. \end{array}$$
(3) The task scheduling scheme is encoded into particles in the HPSO algorithm, and the population is initialized by a chaotic system and opposition learning method to generate a specified number of particles, and the fitness of each particle is calculated. The calculation method is shown in the following formula:
  (16) $$\begin{array}{c} \text{Fit}=\frac{1}{\text{complete Time}}, \end{array}$$
  where complete time indicates the longest running time of all virtual machines.
(4) Dynamically update the inertia weight according to the number of iterations, update the speed and position of each particle, and record the best historical position of each particle and the global best position of the population.
(5) Calculate the fitness value variance corresponding to all particles in the population. If the variance is less than the threshold set by the HPSO algorithm or the number of iterations is greater than the maximum number of iterations set by the algorithm, the algorithm stops the iteration and outputs the smart home control and management task scheduling scheme. Otherwise, the algorithm returns to step 4 to continue searching for the optimal solution.

## 3. Experimental Design

### 3.1. Function Test

The smart home control management client is developed in Java and runs under an Android system. Therefore, the functional test of this method mainly focuses on Android devices. The running environment table of APP client is shown in Table 4.

In the equipment control, the control functions of different equipment are different and limited to space. Taking the lighting control as an example, the test process of equipment control is shown in Table 5:

Scenario management is mainly set to facilitate user operation. By operating scenario management, users can operate multiple devices at the same time, facilitate user operation, and liberate the user operation process, so that users do not have to operate multiple devices at the same time. The test process of scenario management is shown in Table 6.

Through the test of main functional device control and scenario management, its functions can operate normally on all Android devices and meet the expected objectives. At the same time, the relevant smart home management control interface can be displayed normally and meet the requirements on all mainstream models and mainstream systems.

### 3.2. Communication Performance Test

Two ZigBee communication modules are selected. Both modules do not add power amplifier. One node is used as coordinator, the other is used as terminal node, the coordinator is used as sender, and the terminal node is connected to the serial port of PC as receiver. Test in the open without other interference, make the coordinator send 1000 data packets each time through the serial port assistant, test 10 times at each distance point, and finally take the average value as the result. The test results are shown in Table 7.

Through the analysis of the test results, it can be seen that the maximum distance of normal communication between the two nodes is 100 m. At this time, the communication situation is very good, and the packet loss rate is 0. When the distance exceeds 100 m, packet loss begins. At the same time, during the test, it is found that the communication situation will be affected by the surrounding environment. When a vehicle or tester calls, the communication situation will be affected, and the antenna must be placed vertically during the test.

Through the previous communication distance test, it is known that when the communication distance between two nodes exceeds 100 m, packet loss begins to occur. If a node is added between the two nodes, the packet loss rate can be reduced by relaying and transmitting information through this node. Three nodes are selected, one of which is used as the coordinator to send data, and the other two are used as routers.

Firstly, the coordinator and one of the routing nodes are placed at a distance of 200 m. It can be seen from the sending packet test that the communication between the two nodes cannot be realized at this time. Then, the second routing node is added to the two nodes, and the meaning is repeated. Delete one of “about” and “about” for 5 tests. The coordinator sends 1000 packets each time, The test results are shown in Table 8.

Through the analysis of the test results, we can know that when the communication distance between two nodes exceeds the normal communication distance, the communication between nodes can be realized by adding routing nodes. Affected by the surrounding test environment, very few packets will be lost.

### 3.3. Application Effect Test

**Figure 12:**
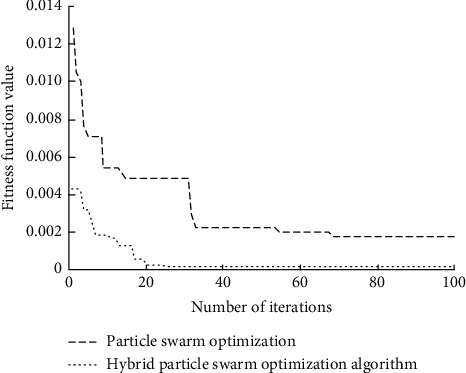
Algorithm convergence comparison.

In order to verify the superiority of the smart home control management task scheduling method based on the hybrid particle swarm optimization algorithm designed in this article, the convergence of the hybrid particle swarm optimization algorithm and the particle swarm optimization algorithm is compared, and the comparison results are shown in Figure 12.

Analysis of the results in Figure 12 shows that with the increase in iteration times, particle swarm optimization achieves convergence only at 70 times, whereas hybrid particle swarm optimization achieves convergence only at 25 times, with faster convergence speed and better comprehensive performance.

**Figure 13:**
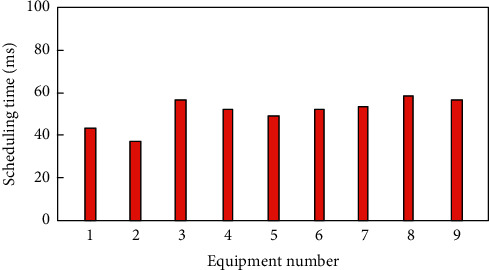
Task scheduling time.

On the basis of the above, use the method to control the audio and video equipment, lighting, curtain control, air-conditioning control, security systems, digital cinema, video server, shadow cabinet system, network home appliances and other equipment, and number of the equipment processing, with 1∼9, said of the equipment control management task scheduling, scheduling time consuming as shown in Figure 13.

According to the analysis of Figure 13, the control and management task scheduling method of smart home based on hybrid particle swarm optimization algorithm takes different time for the control and management task scheduling of different devices. Among them, the management task scheduling time for equipment 6 is the highest, which is 59 ms, while the control and management task scheduling time for equipment 2 is the lowest, which is 36 ms. The overall task scheduling time of smart home management and control task is always less than 59 ms, which shows that the task scheduling time of this method is shorter and more efficient

**Figure 14:**
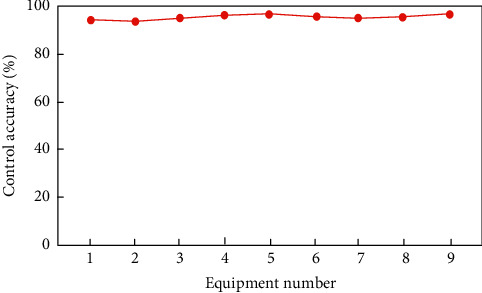
Control accuracy rate.

On this basis, the accuracy of the proposed method for smart home control is verified. The control accuracy refers to the ratio between the number of controlled events and the control password after executing the control password many times. The results are shown in Figure 14.

Analysis of the data in Figure 14 shows that the accuracy rate of the smart home control management method based on big data analysis is always above 93% for smart home control, among which the control accuracy rate of equipment 2, namely, lighting system, is the lowest at 93%. For equipment 5, that is, the control accuracy of security system is the highest, 98%, indicating that using this method can achieve control and management of smart home.

**Figure 15:**
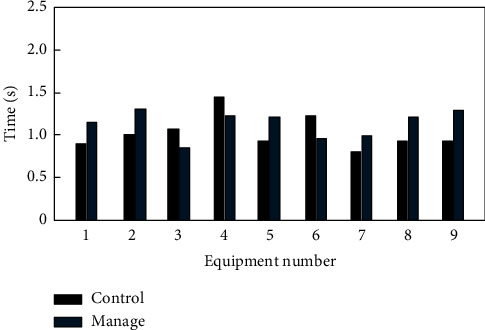
Controlling and managing time.

Comprehensively verify the method in this article for smart home control and management time, and the comparison results are shown in Figure 15.

According to the analysis of Figure 15, the smart home control management method based on big data analysis has certain differences in the management and control time of different devices. The maximum value of the smart home control time of this method is 1.4 s and the minimum value of the smart home control time of this method is 0.8 s; the maximum value of smart home management time of this method is 1.3 s and the minimum value of smart home management time of this method is 0.8 s, which shows that the smart home control management time of this method is shorter and more efficient

## 4. Conclusion

In today's society with the continuous development of science and technology and the material life-level unceasing enhancement, people has higher requirement to the household life environment. The experimental results show that the method can improve the equipment control and management situation and communication performance; the algorithm has a better convergence; smart home control task scheduling time is always under 59 ms, smart home control accuracy always stay above 93%, smart home control time shorter, to explain the practical application of this method is better. It can lay a solid foundation for the further development of smart home field.

## Figures and Tables

**Table 1:** MQ2 detection range.

Name of detected substance	Detection concentration range

Liquefied gas and propane	100 ppm∼10000 ppm
Butane	300 ppm∼5000 ppm
Methane	5000 ppm∼20000 ppm
Hydrogen	300 ppm∼5000 ppm
Alcohol	100 ppm∼2000 ppm

**Table 2:** DHT11 pin description.

Pin	Name	Notes

1	VDD	The supply voltage is 3∼5.5 V DC
2	DATA	Single bus, serial data
3	NC	The empty pin should be suspended
4	GND	Grounding terminal or negative terminal of power supply

**Table 3:** Characteristic parameters of the infrared sensor.

Property name	Characteristic parameter

Working current	The range is 4.4 V∼20 V
Quiescent current	<50 *μ*A
Level output	High level 3.3 V, low level 0 V
Trigger mode	Repeat trigger/nonrepeat trigger
Delay time	5∼200 s, adjustable
Blocking time	The default is 2.5 s, and the adjustable range is several to tens of seconds
Overall dimension	32 mm *∗* 24 mm
Induction angle	Cone angle less than 100 degrees
Working temperature	−15°C∼+70°C
Sensing lens size	The default diameter is 23 mm

**Table 4:** Client running environment.

Client	Android model	Operating system

Host 1	Millet	Android 4.0
Host 2	Huawei	Android 4.3
Host 3	Samsung	Android 5.0

**Table 5:** Equipment control test process.

Test function	APP test process	APP test results	Backstage

Equipment control	(1) Enter the electrical management interface	All lights on	The background receives the general opening instruction and sends the instruction to the equipment
	(2) Select intelligent lighting function		
	(3) Select general opening		

Equipment control	(1) Enter the electrical management interface	All lights are off	The background receives the general closing instruction and sends the instruction to the equipment
	(2) Select intelligent lighting function		
	(3) Select general off		

Equipment control	(1) Enter the electrical management interface	TV wall lights off	The background receives the TV wall closing instruction and sends the instruction to the equipment
	(2) Select intelligent lighting function		
	(3) Select TV wall off		

Equipment control	(1) Enter the electrical management interface	Atrium lights on	The background receives the atrium light on command and sends the command to the equipment
	(2) Select intelligent lighting function		
	(3) Select the atrium light		

**Table 6:** Scenario management test process.

Test function	APP test process	APP test results	Backstage

Scenario management	(1) Enter the main menu	All preset home scene functions are turned on, such as air-conditioning, and curtain	The background receives the general opening instruction and sends the instruction to the equipment
	(2) Select scenario function		
	(3) Choose to go home		

Scenario management	(1) Enter the main menu	All preset home mode functions are turned on, such as turning off lights, and curtains	The background receives the general closing instruction and sends the instruction to the equipment
	(2) Select scenario function		
	(3) Select home mode		

Scenario management	(1) Enter the main menu	The preset one key deployment function is all turned on, the camera is turned on, the camera content can be viewed anytime and anywhere, and the user will be prompted if there is a warning during the operation of the camera	The background receives the TV wall closing instruction and sends the instruction to the equipment
	(2) Select scenario function		
	(3) Select one key deployment		

Scenario management	(1) Enter the main menu	The preset one button disarm function is turned on, and the camera is turned off	The background receives the atrium light on command and sends the command to the equipment
	(2) Select scenario function		
	(3) Select one click disarm		

**Table 7:** Communication distance test.

Serial number	Test distance	Packet loss number	Packet loss rate

1	30	0	0
2	70	0	0
3	100	0	0
4	110	8	0.8%
5	120	10	1.0%
6	150	132	13.2%

**Table 8:** Routing test.

Serial number	Packet loss number	Packet loss rate

1	0	0
2	0	0
3	1	0.1%
4	0	0
5	2	0.2%

## Data Availability

The raw data supporting the conclusions of this article will be made available by the authors, without undue reservation.
